# Book Review

**Published:** 1950-01

**Authors:** 


### Reviews of Books

**Cystoscopy and Urography.** By J. B. Macalpine, D.Sc., F.R.C.S. Third Edition. Pp. 570. Illustrated. Bristol: John Wright & Sons Ltd. 1949. Price 63s.—This scientific treatise is one of which British surgeons the world over may be proud. It is a model of exposition in every aspect and a magnificent legacy from a doyen of urology. The faultless accuracy both in the illustrations and the written word reflects the mind of the author. Nothing is omitted; all is treated with dignity and precision. Particularly valuable are the opening chapters which deal with the complex armamentarium of cystoscopy and leave no excuse for incompetence on the part of the surgeon or staff. Illustrative cases are quoted maintaining a clinical interest throughout the book and the controversial aspects of uro-pathology are dwelt upon. This work provides the present-day cystoscopist with a most comprehensive and complete guide.

**Introduction to Surgery.** By Rutherford Morison, M.D., F.R.C.S., D.C.L., LL.D. and C. F. M. Saint, C.B.E., M.D., M.S., F.R.C.S., F.R.A.C.S. Fourth Edition. Pp. 330. Illustrated. Bristol: John Wright & Sons Ltd. 1948. Price 42s.—A fourth edition of this book, after an interval of twelve years, is very welcome. The object of the book is to emphasize the general principles on which surgery is built, and to enable the student to work out for himself the problems he will meet with in his icinical cases. Many of the classical methods of treatment are described ln more than one association, e.g. the use of B.I.P.P., in the treatment of abscesses. The recent student may rarely havc seen some of these accepted procedures, but they are methods that have given excellent results in the past, and should still hold their place in modern surgery. Throughout the book the clinical experience of the authors forms the basis for treatment; the chapter dealing with appendicitis and peritonitis stands out. Immediate operation is advised in all cases of appendicitis, irrespective of the duration of the disease, but in peritonitis a conservative line is recommended. The use of sulphonamide drugs and penicillin is referred to repeatedly, but stress is not put on the indications and contra-indications. Blood transfusion is rather briefly dealt with and little is said of its dangers. The 304 illustrations all illustrate the clinical conditions in an excellent manner. This book can be strongly recommended to the medical student; it should also appeal to the surgeon and the teacher.

**Bedside Diagnosis.** By C. M. SEWARD, M.D., F.R.C.P. Pp. xii, 372. Edinburgh: E. & S. Livingstone Ltd. 1949. Price 17s. 6d.—No clinician ever feels satisfied with a book on diagnosis, for no book on medical diagnosis can reflect more than the conventions of diagnostic method. Dr. Seward, writing plainly and directly about the art of diagnosis as he sees it, succeeds as well as, if not better than, other clinicians who have attempted the like. It is a difficult book to read right through; but used as a systematic key, it will prove a stimulus and source of new thought both to the practitioner and to the student. The characteristic and most valuable feature of the book is the presentation of physiological fact as a preliminary to diagnosis. To the practitioner, puzzled by a problem in diagnosis such as, for example, a patient with lateral abdominal pain, this book gives a clear insight into anatomical sources and physiological mechanisms of pain from this quarter, and also an estimate of the likely causes and the special features of each of them. This method is applied to pain in all parts of the body, to special symptoms such as haematemesis and haemoptysis, and to symptom complexes such as anaemia, fever and debility.

The final-year student, wishing to tidy the litter of hastily acquired empirical knowledge in his overtired brain, will find in this book a clear and helpful source of reference. There are a few minor errors of fact; and very occasional lapses into unsubstantiated dogmatism. One example of the latter is the bald statement on p. 78 that the proximate cause of pain in nervous dyspepsia is pylorospasm. The cover and binding of the book are neither stout nor aesthetically pleasing; but the printing is clear and there are some useful diagrams.

**Proceedings of the Ninth International Congress on Industrial Medicine, London, 1948.** Pp. 1115. Illustrated. Bristol: John Wright & Sons Ltd. 1949. 60s.—This book contains the papers read at the Congress held in London in the summer of 1948 when contributions were made to the whole field of industrial medical and nursing practice. There are articles on work and skill, incentives and absenteeism, dermatology and ophthalmology, and the hazards and control of radiant energy. There are excellent accounts of pneumoconiosis and other lung diseases, of occupational tumours of the bladder, and of atmospheric pollution with special reference to the dust problem in mining. Proposals are made for the better training of the industrial medical officer and the integration of occupational health with other health services. All the contributors are experts in their respective fields. The book is well produced and sufficiently illustrated. It contains something of interest to may clinicians and will be often consulted by those whose chief interest is the prevention of disability.

**Synopsis of Obstetrics and Gynaecology.** By Aleck W. Bourne, F.R.C.S. Tenth Edition. Pp. 530. Illustrated. Bristol: John Wright & Sons Ltd. 1949. Price 21s.—The dogmatic teaching in this book makes it very valuable to the student: its completeness also makes it of value as a book of reference. As usual we are told that a persistent occipito-posterior may be due to insufficient flexion; also, as in most text-books, the operator is told to rotate the occiput forward. No mention, however, is made of flexion in order to make rotation easy.

**Conduct of Life Assurance Examinations.** By E. M. Brockbank, M.B.E., M.D., F.R.C.P. Third Edition. Pp. 176. London: H. K. Lewis & Co. Ltd. 1949. Price 12s. 6d.—The last edition of this book was reviewed two years ago so that there is little to add. Several improvements have been made. The various forms of insurance and pension schemes are dealt with in considerably more detail. The section on blood pressure has been revised, as has also the one on glycosuria. The author has also made some statements about the undoubted increase in deaths from carcinoma of the bronchus. It remains a very sound account of various forms of examination for life assurance and guide to the interpretation of abnormal findings. The fact that a new edition has been called for in two years shows that it has been considerably used by members of the profession.

**A Synopsis of Medicine.** By Sir Henry Tidy, K.B.E., M.D., F.R.C.P. Ninth Edition. Pp. 1264. Bristol: John Wright & Sons Ltd. 1949. Price £1 10s. 0d.—In this ninth edition will be found much that is new and many revisions. The new articles deal with two groups: old diseases included for the first time and new diseases or syndromes. The author qualifies the latter by describing them as " so-called " new diseases, and mentions in his preface, Acute Infectious Lymphocytosis, Temporal Arteritis, Acute Cortical Necrosis of the Kidney, and the influence of Maternal Rubella on Congenital Defects. Of this influence of Rubella a wise caution is expressed, " some evidence exists that some other virus infections may have similar action during pregnancy ". An open mind on the problems of mental and cerebral defects is more than ever necessary. Much new work on Malaria is included, and the re-arranged and re-written article on Typhoid Fever throws fresh light on an old disease. Yet with all that has been added the size of the volume has only increased by four pages. There is a freshness and youth about this ninth edition that makes it hard to believe that Tidy's *Synopsis of Medicine* is now in its thirtieth year. The printing and production is better than ever—the paper in particular is of a better quality than in some of the later reprintings. No doubt this is the result of the new printing works of the Stonebridge Press, recently brought into use by John Wright & Sons at Brislington, Bristol.

**Medical Annual, 1949.** Edited by Sir Henry Tidy, K.B.E., M.D., F.R.C.P. and A. Rendle Short, M.D., B.S., B.Sc., F.R.C.S. Pp. 448. Illustrated. Bristol: John Wright & Sons Ltd. 1949. £1 5s. 0d.—The sixty-seventh issue of this annual maintains the high standard of its fore-runners. Foremost among the articles must be ranked that on Malaria which embodies much new light on the life-cycle of malarial parasites: it is a matter for regret that the Malaria Research Unit at Cairns in Queensland is now closed. Next to Malaria comes the recent work on Bilharziasis: it is surprising to read that this disease is the most extensive in distribution after Malaria. There is an important article on the care of chronic and aged sick by Dr. Marjory Warren, the English pioneer in this study. Occupational health and industrial disease continue to attract attention and are well discussed. Advances in the treatment of megasoblastic anaemia continue and the new cobalt containing vitamin, known as B12, is fully described. Surgical articles are no less interesting especially gastric and thoracic. The section on legal decisions and legislation is well worth reading: especially a decision dealing with a local partnership case in which the judge described the whole wording of the partnership's covenant as so hopelessly vague as to render the restrictive clause void. Evidently the pitfalls of medical covenants and agreements are not always avoided by the most experienced lawyers.

**Rational Medicine.** By J. W. Todd, M.D., M.R.C.P. Pp. viii, 378. Bristol: John Wright & Sons Ltd. 1949. Price 25s.—This is an unusual and stimulating book. The author writes critically on the interpretation of physical signs and symptoms and on the general principles of medical treatment. Hardly a page is turned before yet another medical myth has been debunked. Perhaps this rather negative attitude may account for the fact that the book is difficult to read consecutively without a feeling of mental exhaustion. However, it contains so much good material and original thought that the book can be earnestly recommended to all doctors, particularly the general practitioner and the consulting physician. They will find that to read a chapter of this book will pleasantly disturb their preconceived ideas, and set free in them thoughts long imprisoned by usage and tradition. The author is particularly to be congratulated on his illustrations of the emotional causes of disease.

**Operative Surgery.** By Frederick C. Hill, M.S., M.D. Pp. xii, 696. Illustrated. New York: Oxford University Press (Geoffrey Cumberlege). 1949. 63s.—This book is beautifully bound, printed on excellent paper and profusely illustrated with clear drawings. A change from many textbooks of operative surgery is the starting of each chapter with a consideration of the pathology of the conditions for which operative procedures will be described, rather than the anatomy of the organs concerned. In his preface, the author states that his book is designed for the intern, resident and less experienced surgeon. Some operations are well described but many are not: and space is wasted in descriptions and illustrations of alternative operations (for example for peptic ulcer). Often no advice is given as to which operation to select. For instance transpleural and extrapleural drainage of subphrenic abscess are described but there is no mention of the now established superiority of the extrapleural route. There are some remarkable omissions. No operation on the sympathetic nervous system is described, and no method of prostatectomy other than suprapubic is mentioned. Admittedly the scope of the book is deliberately limited but such restraint is inconsistent with a detailed description of total gastrectomy and of pneumonectomy. Some of the advice is, even allowing for individual opinion, unsound. The less experienced surgeon may be seriously misled by reading that excision is the only treatment of value for ganglion of the wrist, that the best treatment for a boil is hot fomentations and incision, and that severed nerves should be sutured immediately whenever possible. Equally misleading is the section on anal fistula, where the final wound is shown as a narrow deep trench instead of a wide flat saucer, and that on anal fissure, where no mention is made of the necessity of dividing the subcutaneous sphincter muscle. A more general criticism is that there is throughout a failure to get down to hard facts, little about the difficult situations one meets and how to deal with them. One looks for guidance but finds only instruction. It is no doubt very difficult to write a good and readable textbook of operative surgery, which accounts for the comparatively few which exist. Though this one must be classed as readable it cannot, in its first edition, be rated as good.

**Mongolism.** By M. ENGLER, M.D. Pp. viii, 208. Illustrated. Bristol: John Wright & Sons Ltd. 1949. Price £1 1s. 0d.—Dr. Engler is not satisfied with the term "Mongolism" and proposes "peristatic amentia" for aetiological reasons. The first part of his book is devoted to a description of the condition—history, geography, pathology, prognosis, and treatment. With these points he deals thoroughly along accepted lines. The second part of the book is devoted to the thesis that this condition is due to the implantation of a normal ovum in uterine mucosa abnormal on account of immaturity, curetting, contraceptives, miscarriage, multiparity or senescence. Those interested in this question will of course examine the author's arguments for themselves. Text, illustrations, index and general production are well up to the high standard so long associated with Wright's.

